# A Study of p63 Immunohistochemical Expression in Benign and Malignant Breast Lesions at a Tertiary Hospital in South India

**DOI:** 10.7759/cureus.74905

**Published:** 2024-12-01

**Authors:** Pavani Velamala, Sunil Kumar Dogga, Naresh Tandyala, Murali Mohana Rao Dhavala, Priyanka Pappala, V R Raja Sekhar Sanuvada

**Affiliations:** 1 Pathology, Great Eastern Medical School and Hospital, Srikakulam, IND; 2 Neurology, Great Eastern Medical School and Hospital, Srikakulam, IND; 3 Preventive Medicine, Great Eastern Medical School and Hospital, Srikakulam, IND

**Keywords:** breast, breast lesion, immunohistochemistry, malignant, myoepithelial cells, p63

## Abstract

Introduction

Breast cancer is one of the leading causes of cancer deaths in female patients. Breast lesions can have various morphological diversities, ranging from benign to in situ to malignant. An important histopathological feature that distinguishes benign and malignant lesions is the presence or absence of the myoepithelial cell layer. The tumor protein p63 has been characterized as a reliable immunohistochemical marker and is uniquely displayed in the myoepithelial cells of breast parenchyma. Hence, it helps distinguish benign from malignant lesions.

Aim

To study the value of the immunohistochemical expression of p63 in the diagnosis of breast lesions, to investigate p63 expression in benign and malignant breast lesions, and to assess if loss of p63 expression is consistently associated with invasive disease.

Materials and methods

About 98 cases of breast disease were studied, of which 86 were subjected to immunohistochemical staining for p63 and analyzed for its expression, and the staining arrangement was interpreted.

Results

Out of 86 cases, there were 46 benign and 40 malignant breast lesions. Immunohistochemical staining for p63 in the benign lesions showed continuous positivity with a score of three in 30 cases and discontinuous positivity with a score of two in 16 cases. In malignant lesions, p63 was not expressed and scored zero in 35 cases and discontinuous positive with a score of one in five cases. The sensitivity, specificity, positive predictive value, negative predictive value, and accuracy of p63 in distinguishing the benign from the malignant lesions was 100%, 87.50%, 90.20%, 100%, and 94.19% respectively.

Conclusion

There is a statistically significant difference in the expression of p63, a specific nuclear marker for myoepithelial cells, in benign and malignant breast lesions. It was consistently positive in benign breast lesions and negative in the majority of malignant breast lesions. Thus, p63 can act as a helpful immunohistochemical marker in categorizing histopathologically-difficult cases into benign or malignant ones.

## Introduction

After lung cancer, breast cancer is the most frequent cause of cancer deaths among women worldwide [1]. It accounts for the maximum morbidity and mortality in women globally and imposes a huge burden on healthcare [2]. The morphologic distinction between benign and malignant (in situ and invasive) diseases of the breast can be challenging, particularly in the setting of core needle biopsy. Although morphology alone can diagnose the majority of breast lesions, significant variation in the interpretation of challenging lesions based on histological analysis has been reported [3]. Immunohistochemical staining can, therefore, help diagnose and determine the prognosis of these lesions [4]. The cells of the ductal epithelium are of three types: luminal, basal, and myoepithelial cells [5]. Different cytokeratins are described in the luminal and basal cell types, whereas myoepithelial cells express basal cell-type cytokeratins, smooth muscle actin, calponin, and p63 [6]. The retention of the myoepithelial layer is often seen in benign and in situ lesions, whereas loss of this layer is considered a diagnostic feature of invasive cancer [7,8]. Werling et al. conducted a comparative analysis of p63 against calponin and smooth muscle myosin heavy chain (SMMHC) and found that p63 is the most specific among the three markers, and could replace calponin and SMMHC [9], as the latter showed an affinity for myoepithelial cells and myofibroblasts [10,11]. p63 is a specific nuclear marker for myoepithelial cells as it neither stains stromal fibroblasts nor vascular smooth muscle cells and can be very helpful in revealing invasion. It is also easily appreciated, even in cytologic preparations [12]. p63 staining was observed around the normal ducts and benign tumors but was absent in invasive carcinomas. It also has a complementary role in distinguishing in situ from invasive lesions [13]. This study aimed to investigate p63 expression in benign and malignant breast lesions and to assess if loss of p63 expression is consistently associated with invasive disease.

## Materials and methods

This cross-sectional study was conducted in the Department of Pathology, Great Eastern Medical School and Hospital, Srikakulam, Andhra Pradesh over one year, from July 2023 to June 2024, after ethical clearance from the Great Eastern Medical College Institutional Ethical Committee (195/IEC/GEMS&H/2024). A total of 98 breast disease cases were collected, in the form of trucut biopsies, lumpectomies, and mastectomies. Clinical details were procured from the patients' histopathology requisition forms and the hospital information management system (HIMS). All cases underwent standard processing and were stained with hematoxylin and eosin (H&E) for analysis.

Inclusion criteria

All incisional biopsies, trucut biopsies, lumpectomy, and mastectomy specimens of both benign and malignant breast lesions were included in the study.

Exclusion criteria

All congenital breast diseases, inflammatory breast lesions, metastatic deposits to the breast, cases with prior treatment, and inadequate biopsies were excluded from the study.

Sample size calculation

The sample size was calculated based on the sensitivity estimation formula, n=Z^2^ - α/2{Sensitivity(1-Sensitivity)}/d^2^ X prevalence, with 20% sensitivity, and 90% prevalence of the outcome in the population [9]. The minimum sample size needed was 36.

Study procedure 

Tissues were routinely fixed in 10% neutral buffered formalin, embedded in paraffin blocks, sectioned at three to five microns, and stained with H&E. They were studied under a light microscope. For positive control of p63 staining, a histological section from a benign prostate biopsy was included in each staining batch. For the negative control, FLEX ready-to-use, monoclonal mouse universal negative control (code IR750; Agilent Dako, California, United States) was used.

The procedure involved heat-induced epitope retrieval with a Tris/Ethylenediamine tetraacetic acid (EDTA) buffer at pH 9.0, followed by inactivation of endogenous peroxidase using aqueous hydrogen peroxide. The samples were then incubated with the primary antibody (mouse anti-human p63 monoclonal antibody, Clone 4A4, Vitro Master Diagnóstica, Madrid, Spain), followed by diaminobenzidine (DAB) chromogen application and counterstaining. All histopathology sections were evaluated, and immunohistochemical staining was performed on 86 cases. The IHC-stained slides of these cases were studied in detail and categorized into benign lesions, and non-invasive and invasive carcinomas.

p63 expression assessment

The intensity of p63 expression was categorized as continuous positive, discontinuous positive, or negative. The extent was quantified by the percentage of positive cells, with scores assigned as follows: zero (negative), one (1-25%), two (26-90%), and three (91-100%). Scores of one and two indicated discontinuous positivity, while a score of three indicated continuous positivity. Based on the p63 immunostaining, sensitivity, specificity, positive predictive value, negative predictive value, and accuracy were calculated. The Fisher's exact test was used for quantitative analysis of data. Mean ± standard deviation (SD) and percentage were used to determine data for statistical analysis by using IBM SPSS Statistics for Windows, Version 20 (Released 2011; IBM Corp., Armonk, New York, United States) and the p-value was calculated. A p-value of less than 0.05 was deemed statistically significant.

## Results

A total of 98 cases were evaluated, of which 12 were excluded. Thus, 86 cases were included in the study. Among these, there were 35 lumpectomy specimens (40.69%), 25 modified radical mastectomy specimens (29.0%), 24 trucut biopsies (27.9%), and two simple mastectomy specimens (2.3%). The patients in the study ranged in age from 18 to 80 years, with the majority of cases occurring in the fifth decade of life, followed by the fourth and the third decades. The average age at presentation was 40.8 years, and the study included 81 females and five males.

Of the total cases analyzed, 46 (53.48%) were benign and 40 (46.51%) were malignant. The most prevalent histological variant among the benign breast lesions was fibroadenoma, followed by sclerosing adenosis and gynecomastia (Table 1).

**Table 1:** Histopathological diagnosis of the various benign lesions

Diagnosis	No of cases (n)	Percentage (%)
Fibroadenoma	24	52.17
Intraductal papilloma	1	2.17
Benign phyllodes	1	2.17
Fibrocystic change	4	8.69
Sclerosing adenosis	5	10.86
Usual ductal hyperplasia	2	4.3
Atypical ductal hyperplasia	4	8.69
Gynecomastia	5	10.86
Total	46	100

**Figure 1:**
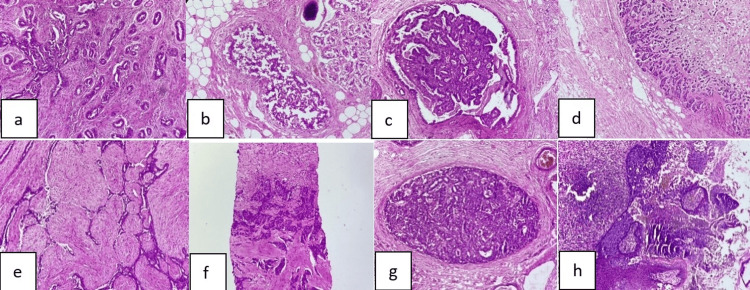
Histopathological pictures of the various breast lesions a) Fibroadenoma shows proliferation of duct and stroma with peri canicular stromal growth (H&E, 40X); b) Benign phyllodes tumor shows a leaf-like epithelial pattern. (H&E, 40X); c) Intraductal papilloma shows a fibrovascular core with overlying layers of myoepithelial and epithelial cells (H&E, 100X); d) Atypical ductal hyperplasia shows epithelial proliferations in cribriform pattern (H&E, 100X);  e) Ductal carcinoma in situ shows proliferating neoplastic cells within the duct (H&E, 100X); f) Metaplastic carcinoma shows areas of chondroid and epithelial differentiation of tumor cells (H&E, 100X); g) Invasive ductal carcinoma shows tumor cells in nests and islands (H&E, 40X); h) Paget's disease of breast shows intraepidermal migration of ductal carcinoma cells (H&E, 100X). H&E: hematoxylin and eosin stain.

In contrast, infiltrating ductal carcinoma was the most common variant among the malignant lesions (Table 2 and Figure 1).

**Table 2:** Histopathological diagnosis of the various malignant lesions

Diagnosis	No of cases (n)	Percentage (%)
Invasive ductal carcinoma	29	72.5
Ductal carcinoma in situ	4	10
Invasive lobular carcinoma	2	5
Mucinous carcinoma	1	2.5
Solid papillary carcinoma	1	2.5
Metaplastic carcinoma	1	2.5
Medullary carcinoma	1	2.5
Paget's disease of breast (underlying invasive carcinoma)	1	2.5
Total	40	100

**Figure 2:**
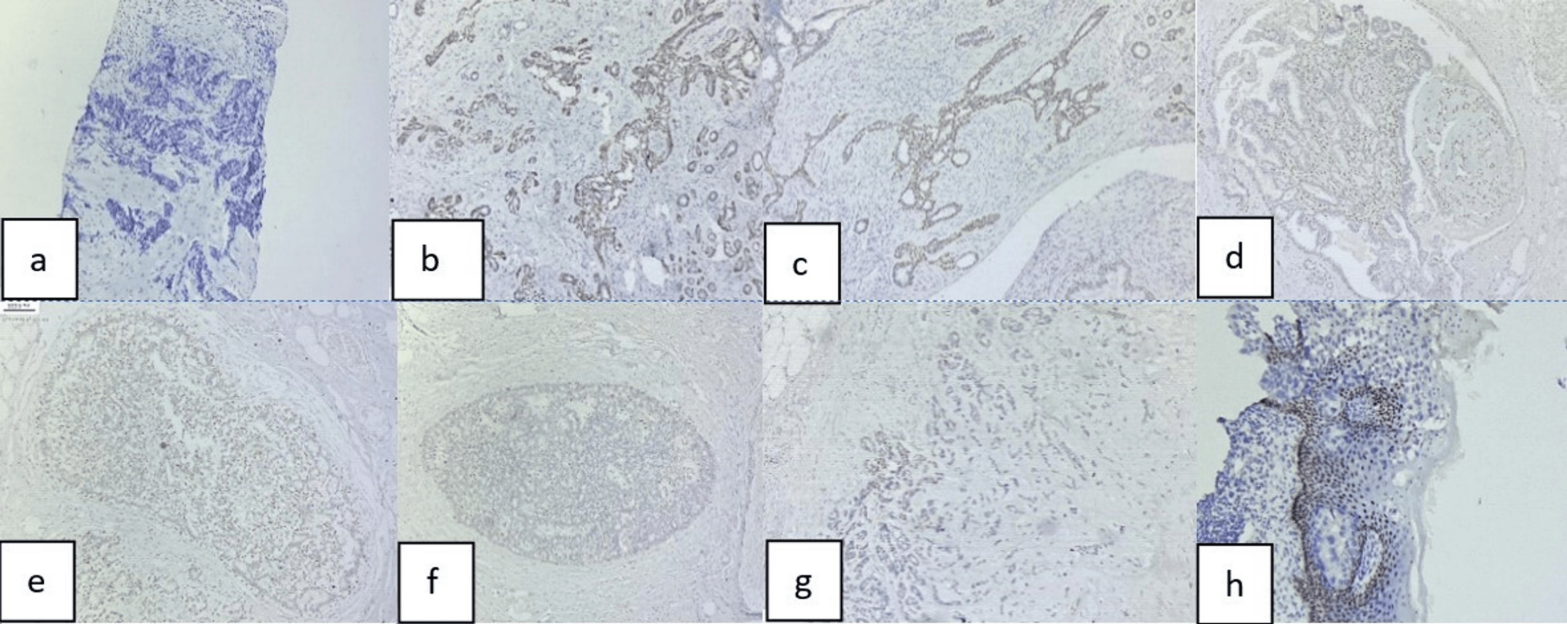
Immunohistochemical expression of p63 in the various breast lesions a) Fibroadenoma shows score three (p63, 40X); b) Benign phyllodes with score three (p63, 40X); c) Intraductal papilloma displaying score three (p63, 100X); d) Atypical ductal hyperplasia exhibiting score two (p63, 100X); e) Ductal carcinoma in situ showing discontinuous immunostaining with score one (p63, 100X); f) Metaplastic carcinoma showing score one (p63, 100X); g) Invasive ductal carcinoma, score zero (p63, 40X); h) Paget's disease of breast showing negative immunostaining for p63 score zero, whereas the normal keratinocytes are positive (p63, 100X).

p63 immunostaining was conducted on all the cases. Among the 46 benign cases, continuous p63 positivity with a score of three was observed in 24 fibroadenomas, four cases of fibrocystic change, one benign phyllodes tumor, and one intraductal papilloma (Table 3). Discontinuous positivity with a score of two was noted in five cases of sclerosing adenosis, two cases of usual ductal hyperplasia, four cases of atypical ductal hyperplasia, and five cases of gynecomastia (Table 3 and Figure 2).

**Table 3:** p63 expression and scoring in various types of benign lesions

Diagnosis	No of cases (n)	p63 expression	p63 score
Fibroadenoma	24	Continuous positive	3
Intraductal papilloma	1	Continuous positive	3
Benign phyllodes	1	Continuous positive	3
Fibrocystic change	4	Continuous positive	3
Sclerosing adenosis	5	Discontinuous positive	2
Usual ductal hyperplasia	2	Discontinuous positive	2
Atypical ductal hyperplasia	4	Discontinuous positive	2
Gynecomastia	5	Discontinuous positive	2

Out of the 40 malignant cases, 29 cases of invasive ductal carcinoma, two cases of invasive lobular carcinoma, one case of mucinous carcinoma, one case of solid papillary carcinoma, one case of medullary carcinoma and one case of Paget's disease (underlying invasive breast carcinoma) showed negative p63 immunostaining (score zero; Table 4). The remaining five cases exhibited discontinuous p63 positivity, with a score of one observed in one case of metaplastic carcinoma and four cases of ductal carcinoma in situ (Table 4 and Figure 2).

**Table 4:** p63 expression and scoring in the various types of malignant lesions

Diagnosis	No of cases (n)	p63 expression	p63 score
Ductal carcinoma in situ	4	Discontinuous positive	1
Invasive ductal carcinoma	29	Negative	0
Invasive lobular carcinoma	2	Negative	0
Mucinous carcinoma	1	Negative	0
Solid papillary carcinoma	1	Negative	0
Metaplastic carcinoma	1	Discontinuous positive	1
Medullary carcinoma	1	Negative	0
Paget's disease of breast	1	Negative	0

Among the 86 cases, all 46 benign cases demonstrated positive p63 expression (scores of two or three), resulting in a 100% positivity rate. In contrast, of the 40 malignant cases, 35 (87.5%) showed no p63 expression (score of zero), while five cases (12.5%) exhibited positive p63 expression (score of one; Table 5).

**Table 5:** p63 expression in the benign and malignant lesions

Cases	Positivity for p63		Negativity for p63		Total	
	Number (n)	Percentage (%)	Number (n)	Percentage (%)	Number (n)	Percentage (%)
Benign	46	100	00	00	46	53.48
Malignant	5	12.5	35	87.5	40	46.51
Total	51	59.3	35	40.69	86	100

A significant difference in p63 expression was observed between the benign and malignant breast lesions, with a p-value of less than 0.00001. p63 was consistently positive in the benign breast lesions, indicating that the immunohistochemical expression of p63 can serve as a reliable marker for distinguishing between benign and malignant breast lesions (Table 6).

**Table 6:** Association of p63 with the benign and malignant lesions p-value <0.05 is considered significant

Category	No. of cases (n) positive for p63 (%)	No. of cases (n) negative for p63 (%)	P value
Malignant	n=5 (12.5%)	n=35 (87.5%)	0.00001
Benign	n=46 (100%)	n=0 (0%)	

Based on p63 positivity and negativity in all the benign and malignant breast lesions, sensitivity was found to be 100%, specificity 87.50%, positive predictive value 90.20%, negative predictive value 100%, and accuracy 94.19% (Table 7).

**Table 7:** p63 positive and negative expression in the benign and malignant breast lesions

	Benign lesions	Malignant lesions
p63 positive cases	46	5
p63 negative cases	0	35
Total	46	40

## Discussion

Breast lesions do not constitute a single entity; they encompass a diverse range of diseases characterized by significant clinical and morphological variability [14]. Invasive carcinomas are treated based on clinical, radiological, and pathological findings. The presence or absence of invasion is an important histopathological feature that helps in its treatment and prognosis [15]. Myoepithelial cells have a tumor-suppressive function by secreting protease inhibitors and releasing tumor-suppressive proteins. The transition of benign lesions to invasive carcinoma involves the loss of myoepithelial cells, which leads to the loss of the tumor-suppressive function [16]. Myoepithelial markers are valuable tools for differentiating invasive carcinoma from benign lesions that share similar morphological features [17]. Invasive carcinomas are characterized by the absence of a myoepithelial layer, which typically encases benign breast ducts [18]. p63 is both a sensitive and specific myoepithelial cell marker, as it is expressed exclusively in myoepithelial cells without cross-reactivity with myofibroblasts [19]. The assessment of invasion in malignant cases on routine H&E staining posed challenges in some core needle biopsies due to peritumoral inflammation-associated fibrosis, which obscured the detection of myoepithelial cells. However, negative p63 staining on immunohistochemistry and clinico-radiological correlation confirmed the diagnosis of invasive carcinoma in these cases [20].

Saini et al. found that most breast cases occurred in the fifth decade of life, followed by the fourth decade, with a mean age at presentation of 37.1 years, which was close to our study findings (40.8 years) [21]. There were 86 cases in the present study, of which 46 (53.4%) were categorized as benign lesions, similar to the findings by Thakkar et al. (54.16%) and Stefanaou et al. (52.63%) [22,23]. The remaining 40 cases (46.51%) were classified as malignant, which aligns closely with the findings of Wang et al. (41.17%) but is higher than the results reported by Stefanaou et al. (36.09%) and Verma et al. (32.4%) [23-25].In our study, fibroadenoma was the predominant benign lesion, accounting for 52.17% of cases, which aligns with existing literature where fibroadenomas represent 46.6% to 55.6% of all benign breast lumps [26,27].

Invasive ductal carcinoma emerged as the most prevalent malignant lesion in our study, constituting approximately 72.5% of all malignant cases, a finding consistent with studies by Verma et al. (87.5%) and Tiwari et al. (84.3%) [25,28]. Notably, all benign tumors in our study tested positive for p63. Conversely, the majority of malignant tumors (35 cases or 87.5%) were negative for p63, while five cases (12.5%) exhibited p63 expression, including four cases of ductal carcinoma in situ and one case of metaplastic carcinoma. Similar findings were reported by Barbareschi et al. who noted p63 positivity in all benign lesions, while invasive breast carcinomas displayed consistent negative p63 staining in 95% of cases [29]. Among the 86 cases in our study, 30 (34.88%) benign lesions were continuous positive p63 with a score of three. While five (5.8%) cases of sclerosing adenosis , two (2.3%) cases of usual ductal hyperplasia, four (4.65%) cases of atypical ductal hyperplasia and five (5.8%) cases of gynecomastia were less continuous p63 positivity with a score of two. Out of the 40 (46.5%) malignant cases, four (10%) cases of ductal carcinoma in situ and one (2.5%) case of metaplastic carcinoma showed discontinuous positive p63 with a score of one, while 35(87.5%) invasive carcinomas showed a negative p63 with a score of zero. These findings were almost similar to Saini et al. [21].

Metaplastic breast carcinoma represents a rare and heterogeneous category of primary breast malignancies, comprising less than 1% of all invasive breast carcinomas. In our study, we identified one case of metaplastic carcinoma that exhibited discontinuous positive p63 immunostaining, consistent with the findings of Saini et al. [21]. Mammary Paget’s disease is a type of breast cancer that is usually associated with underlying malignancy of the breast. In this condition, there are atypically large cells with abundant, pale-staining cytoplasm, which sometimes mimicked keratinocytes. However, these Paget’s cells were negative for p63, in sharp contrast to the surrounding keratinocytes [29]. This finding was consistent with our case, where Paget's cells were stained negative for p63 against the background of p63-positive keratinocytes (control).

The identification of a peripheral rim of myoepithelial cells is essential in the differential diagnosis of breast lesions, especially when working with limited core biopsy samples. This characteristic feature is valuable because the loss of p63 expression has been linked to the progression of ductal breast carcinoma, indicating that p63 immunostaining plays a critical role in confirming the presence of invasive growth patterns.

In the present study, p63 demonstrated high sensitivity and specificity for diagnosing benign breast lesions, consistent with findings by Barareschi et al., Reis-Filho et al., and Cheung et al. [3,16,30] who reported a sensitivity of 100% and a specificity of 95%. Therefore, p63 proves to be a highly reliable myoepithelial marker and could be effectively incorporated into immunohistochemical panels to aid in the identification of myoepithelial cells in challenging breast lesions. When comparing p63 immunostaining results between benign and malignant breast lesions using Fisher’s exact test, a statistically significant difference was observed, suggesting that p63 is a reliable marker for differentiating between benign and malignant lesions.

Our findings emphasize the utility of p63 expression in identifying myoepithelial cells in ambiguous breast lesions, in distinguishing various complex epithelial breast lesions, and also in challenging core biopsies. The results further imply that a loss of p63 expression is associated with the invasive progression of breast carcinoma. Consequently, p63 immunostaining may assist in differentiating invasive ductal carcinoma from ductal carcinoma in situ or atypical ductal hyperplasia, thereby aiding in clinical decision-making and ensuring appropriate therapeutic interventions.

The findings of this study must be interpreted after considering several limitations. First, the small sample size may not provide a fully representative view of the general population, potentially limiting the broader applicability of the results. Additionally, variability in p63 expression, intensity, and staining patterns observed in this and other studies suggest that p63 may have complex molecular roles in tumorigenesis, warranting further investigation. Furthermore, differences in staining protocols and interpretation criteria across studies can lead to inconsistent results.

To deepen our understanding of p63 expression across various tumor subtypes and normal tissues, future research should involve a larger and more diverse sample set examined under rigorously standardized conditions. Although large immunohistochemical panels have been used in differential diagnosis, there is still no consensus on the most sensitive and specific antibodies or optimal combination of markers. This ambiguity points to an ongoing need for novel markers and refined diagnostic tools to improve accuracy and reliability in distinguishing between benign and malignant lesions.

## Conclusions

In our study, the pattern of p63 expression was examined across a total of 86 cases. A positive correlation was observed between histopathological features and p63 scoring in all lesions, yielding 100% sensitivity, 87.50% specificity, 90.20% positive predictive value, 100% negative predictive value, and 94.19% accuracy. Among benign lesions, non-proliferative cases demonstrated continuous positivity, while proliferative lesions exhibited less consistent positivity for p63. Premalignant lesions showed minimal positivity and most malignant lesions were devoid of p63 staining, with a few exceptions.

p63 expression proves to be beneficial for diagnosis in challenging trucut biopsy specimens preoperatively and in excisional lumpectomy specimens postoperatively. This study concludes that p63 is a highly valuable immunohistochemical marker for cases that are difficult to classify based solely on histomorphological features.
