# Correction: A Study of p63 Immunohistochemical Expression in Benign and Malignant Breast Lesions at a Tertiary Hospital in South India

**DOI:** 10.7759/cureus.c235

**Published:** 2025-08-11

**Authors:** Pavani Velamala, Sunil Kumar Dogga, Naresh Tandyala, Murali Mohana Rao Dhavala, Priyanka Pappala, V R Raja Sekhar Sanuvada

**Affiliations:** 1 Pathology, Great Eastern Medical School and Hospital, Srikakulam, IND; 2 Neurology, Great Eastern Medical School and Hospital, Srikakulam, IND; 3 Preventive Medicine, Great Eastern Medical School and Hospital, Srikakulam, IND

The Ethics Statement and Conflict of Interest Disclosures section of this article has been corrected to indicate that this study did involve human subjects or tissue.

